# Real-Time Wavefront Sensing at High Resolution with an Electrically Tunable Lens

**DOI:** 10.3390/s23156651

**Published:** 2023-07-25

**Authors:** Ricardo Oliva-García, Carlos Cairós, Juan M. Trujillo-Sevilla, Miriam Velasco-Ocaña, José Manuel Rodríguez-Ramos

**Affiliations:** 1 Wooptix S.L., 38204 La Laguna, Spainmvelasco@wooptix.com (M.V.-O.); jmramos@wooptix.com (J.M.R.-R.); 2 Department of Basic Medial Scienes, University of La Laguna, 38204 La Laguna, Spain; ccairosb@ull.edu.es

**Keywords:** wavefront sensing, phase camera, electrically tunable lens, Shack–Hartmann

## Abstract

We have designed, assembled, and evaluated a compact instrument capable of capturing the wavefront phase in real time, across various scenarios. Our approach simplifies the optical setup and configuration, which reduces the conventional capture and computation time when compared to other methods that use two defocused images. We evaluated the feasibility of using an electrically tunable lens in our camera by addressing its issues and optimizing its performance. Additionally, we conducted a comparison study between our approach and a Shack–Hartmann sensor. The camera was tested on multiple targets, such as deformable mirrors, lenses with aberrations, and a liquid lens in movement. Working at the highest resolution of the CMOS sensor with a small effective pixel size enables us to achieve the maximum level of detail in lateral resolution, leading to increased sensitivity to high-spatial-frequency signals.

## 1. Introduction

A phase camera is an instrument that can simultaneously measure intensity and wavefront aberrations of the incoming light. In general, a phase camera is a combination of a CCD (charge-coupled device) or CMOS (complementary metal-oxide-semiconductor) sensor with a special optical arrangement or a wavefront sensor, and a computer system to translate data to phase maps, intensity maps or a combination of both.

Wavefront phase sensors were first developed to measure the aberration of an optical wavefront produced by an imaging system or other surrounding information acquired by the system [1]. They were developed mainly, and independently, in two fields as different as astronomy [2,3] and ophthalmology [4,5], where, in the first case, they correct the wavefront aberrations produced by the huge telescopic lenses and changes in composition, and in the second, conditions in the atmosphere and aberrations of the human vision system.

Shack–Hartmann (SH) wavefront sensors are widely used in many applications [3,6]. They fit a lenslet array between the camera sensor and the sample, and ultimately measure a set of discrete slopes, so the lateral resolution is limited to the number of lenslets. SH wavefront sensors became the gold standard, due to their simplicity, precision and wide dynamic range and, above all, because these sensors are principally used in adaptive optics (AO) in telescopes [7], where their main drawback, the low resolution, is not a serious problem, since the resolution of these systems is currently limited, not by the resolution of the sensor, but by the resolution of the corrective elements, whether deformable mirrors (DMs), spatial light modulators (SLM), or other corrective elements [8,9].

Interferometric methods have been commonly used to retrieve the wavefront phase of an optical field, although they are not normally considered wavefront sensors, due to the use of a laser and an aberrated reference beam [10,11]. These characteristics limit their applications, especially in fields such as ophthalmology or microscopy, where laser phototoxicity limits either the sensor exposure time or the laser power.

In recent years, many wavefront sensing techniques have emerged as alternatives to the SH system. Some are evolutions of the SH, which use some diffractive element between the sensor and the sample, and present improvements in accuracy [12,13], speed [14], dynamic range [15,16,17,18], or lateral resolution [19,20]. These sensors are still limited in the latter aspect, as they cannot reach full sensor resolution and have other drawbacks typical of an SH sensor, such as low-frequency phase detection, without the possibility of extracting high variations inside the wavefront.

Deterministic or iterative mathematical techniques, phase imaging [21,22] or curvature sensing [23], for instance, can provide wavefront estimation. In these techniques, lateral resolution is limited only by the resolution of the images used to calculate wavefront measurements [24]. The most common approach, used specially in microscopy because it is limited to short defocused distances, is based on the transport of intensity equation (TIE) [21]. These wavefront sensors suffer from linearity problems and narrow dynamic range, and their applications to video recording are limited, because these sensors take multiple images to obtain one phase map.

As wavefront sensors have evolved technically, so have their fields of application. The characterisation of lenses [25], lasers [26], ophthalmic diseases such as keratoconus [27,28], microscopy [29] or medicine [30] are just some of the current and future applications of wavefront sensors. In all these applications it will be convenient to use the highest possible resolution within the largest possible field of view, since in terms of computer vision we have moved from determining the low frequencies required for atmospheric correction to determining high frequencies, which are necessary to elucidate small details within an image. Despite the researcher efforts, the notable evolution of many types of wavefront sensor that have been described above, and the growing number of applications, there are a relatively reduced number of commercial instruments.

We have developed a compact real-time high-resolution phase camera that is able to recover the wavefront phase distortions and the amplitude of an optical field simultaneously. It is a combination of a deterministic wavefront phase sensor that obtains the information from two symmetrically out-of-focus images and an electrically tunable lens (ETL), which allows minimizing capture time and avoiding a mechanical system that moves the camera or the sample. In our technique, we obtain wavefront phase information from defocused images in symmetrical planes before and after the focal point, as was proposed in the original work of Roddier in 1988, with the introduction of the curvature wavefront sensor [31], which avoided using diffractive elements or lenslets. The main innovation in our setup is the placement of the ETL before the Fourier plane, in the pupil plane, which enables us to reduce the setup size, unlike other state of the art works [32] which place it in the Fourier plane. It simplifies the current state-of-the-art setup, which often requires additional control electronics and physical separation of the lens from the camera, and it involves adapting a camera with a fixed lens, along with ETL, specifically designed to capture intensity images.

## 2. Materials and Methods

Our approach consists of a common intensity imaging system combined with an ETL, an optimized optical arrangement and a Jetson AGX microcontroller to manage the electronics, obtain the high frame rate data, and process the outputs with low power consumption. This measuring procedure needs a lens relay to capture around the phase object with a collimated light beam. The most common approaches that captures out-of-focus images use external translation stages to move either the sensor or the object, which requires a high amount of time to stabilise the system and capture both images. This capture system was first designed as an ophthalmologic device [28], using a prism system and one sensor to capture multiple images at the same time. In the presented approach, an ETL was used to enhance the capture process, but the approach requires image processing to solve the aberrations and magnification produced by the lens.

**Figure 1:**
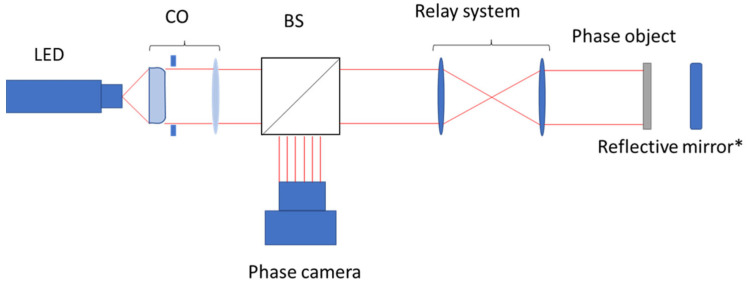
Experimental set-up showing a diagram of the optical system. CO is a collimator system made with an objective and a pinhole in front of the LED. BS refers to the beam splitter, which encompasses the relay system and an optional reflective mirror in cases where the phase object is transparent. * The reflective mirror should be added for transparent phase objects.

The system consists of a wavefront phase image sensor [28,33], which employs a lens relay to reverse the object space and captures two out-of-focus images on either side of the pupil plane, as depicted in Figure 1. This approach eliminates the need for mechanical translation or prism stages, by utilizing an ETL. This methodology provides the capability of modifying the propagation distance rapidly and automatically, enabling real-time measurements. The potential of merging different images obtained at various propagation distances using fast focus switching holds promise [34], and will be explored in future studies to fully leverage the advantages of the system equipped with an ETL. The lack of physical displacement needs image corrections to account for the focal length variations, along with a comprehensive characterisation of the ETL to control defocused distances. This process is crucial to ensure quantitative and accurate measurements.

A lens with a focal length of 30 mm was placed on top of the ETL to enhance the details of the phase object, resulting in a fivefold increase. The relay system is composed of a 150 mm and a 75 mm lenses, augmenting by a factor of 2. The light is located at 25 cm from the BS, and the phase object at over 8 cm in front of the 75 mm lens. Finally, the camera is placed in front of the BS at 12 cm, which is the focal combination that provides the centre of the range for varying the ETL. The telecentric system is common hardware which is added to an ETL optical setup [32], generally used to correct the magnification produced by the focal shift. Our configuration avoids the use of this element, simplifying the mechanical design by solving the problem through a computational approach using the method developed specifically for this work, which is described below.

The phase camera was positioned in front of the beam splitter to ensure that the pupil plane was at the midpoint of the maximum range available with ETL. This allowed for an increased range of defocus distances within the maximum limits of the ETL, which is not necessarily the median of the allowed voltage.

We designed a case holder to incorporate the necessary electronics for cooling the sensor, avoiding temperature effect on dark noise. The holder employs a common Peltier cell with a heat sink, and it is powered using a modified power supply. The design was built using a 3D printer.

**Figure 2:**
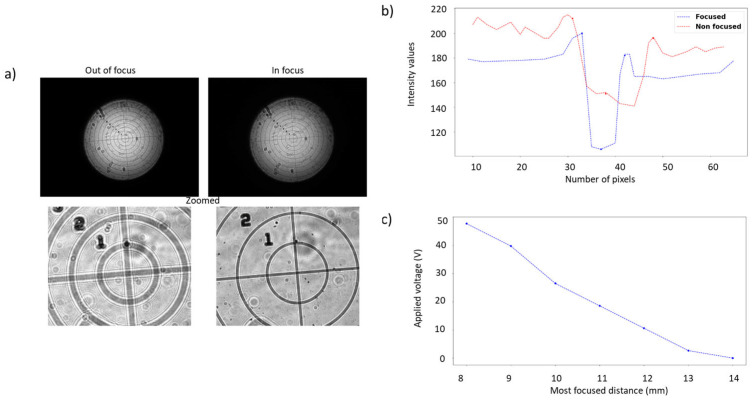
Procedure to calibrate defocus distance (Δz) with the ETL. (**a**) Images with the calibration target in and out of focus. (**b**) Thickness characterisation of multiple-focus images to detect the distance of the object to the camera. (**c**) Calibration comparing most-focused distance with applied voltage.

In our approach the propagation distance depends on the focal movement of the ETL. In the lens selected [35], the mechanical system provides an application-specific integrated circuit (ASIC) to control the focus actuator, with a 10 bit digital-to-analog converter (DAC). Our method to calibrate the focus distances uses a concentric circle reticule target [36] to detect the most focused value on each DAC position, moving a stage on the object side to obtain the distances. Each image was captured every 500 µm in the linear stage. The procedure captures each 1.60 V from 0 V to 60 V, obtaining the characterisation curve. Using the Laplacian kernel [37] and the Radon transform [38] makes it possible to find the maximum variance over the oriented axis of the target, obtaining the maximum variability to decide the correct focus. Figure 2 presents a selection of images with different focuses, showing the variability of the high frequencies when the lens is well focused. Before the calibration, a pupil plane must be chosen, using the lens datasheet, to obtain the central position in distance, not in voltage.

The algorithm finds the minimum thickness of the lines across the oriented angles, as shown in Figure 2b. After the algorithm execution, it is possible to fit a polynomial relation of the voltage-versus-propagation distance, to use this method as a quantitative one, as shown in Figure 2c.

Magnification due to changes in focus distance must be corrected. Our proposal requires the calibration target to obtain the sensor-acquired dimension differences; the magnification is calculated with the size of each target, as in Equation (1),

(1) $$M_{v1,v2}=\frac{T_{v1}}{T_{v2}}$$

where *M* is the magnification factor, *T* the target size and *v*1 and *v*2 the voltage at each DAC. Target size is obtained using the intensity information. In this case, the target is circular, so the diameter of each circle is chosen as *T*, each value of *v* determines the distance in the object space, and the medium point is chosen to locate the pupil plane. To avoid intensity value manipulation, the algorithm always chooses the smallest shape to expand without modifying the values obtained by the sensor. By extracting both shapes from the target, it is possible to obtain the different number of steps needed to adjust the size, as shown in Equation (2),

(2) $$D=\frac{T_{\min T_{v1},T_{v2}}*\left(\left|T_{v2}-T_{v1}\right|\right)}{T_{\min T_{v1},T_{v2}}}$$

where *D* represents the final difference between the two circular pupils. The minimum diameter is chosen to expand the intensity image, ensuring that the information captured by the sensor is not lost.

**Figure 3:**
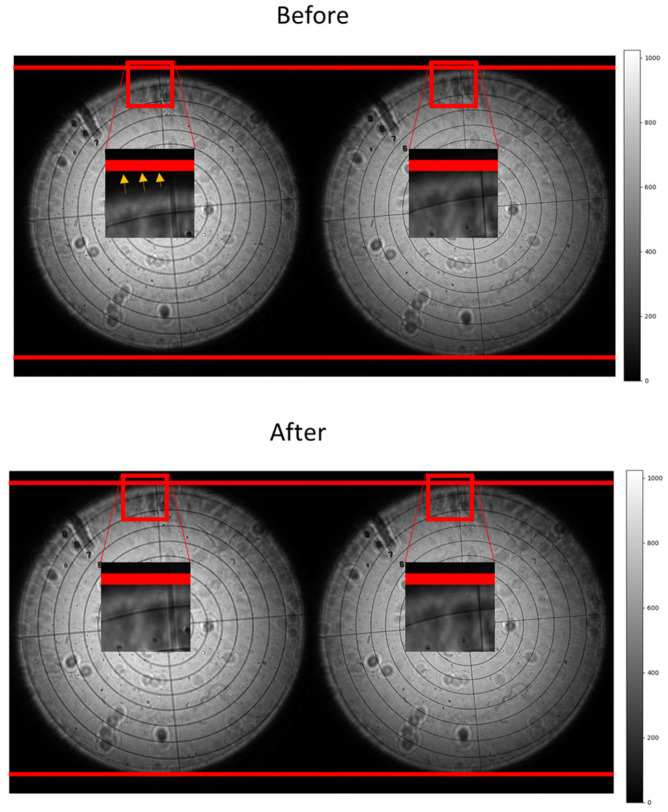
Images captured with symmetric distance from the pupil plane. **Top**: Raw images obtained from the sensor, presenting magnification due to the focal changes. **Bottom**: Magnification corrected after the application of Equations (1)–(3). Red lines represent the pupil size, and the yellow arrows define how the small pupil expands to the other.

With the difference between the images, the step (*S*) for generating the grid is shown in Equation (3),

(3) $$S=\frac{Shape\left(min\left(I_{v1},I_{v2}\right)\right)}{D}$$

where a linear regression is characterised using *N* neighbours to fill the intermediate values; *I* is the intensity image and *v* each voltage, assuming that the images are square. By obtaining the trend in both axis *X* and *Y*, the medium values without information are filled. The idea behind the procedure is to enlarge the image without losing the information captured by the sensor. The intermediate step displays a grid of the S-by-S-sized images, filling the intermediate values with neighbours, so that a common interpolation, such as nearest neighbour or linear interpolation, is not necessary. Figure 3 shows the images before and after the correction.

The last step to obtain the phase map with our approach consists of capturing two intensity images around the pupil plane with an equal defocus, Δz, on each side. With both images, the phase gradients are recovered along two orthogonal directions. Δz must be chosen according to the phase object we are measuring, in order to maximise the frequencies that should be extracted from the object [21,39]. The optical setup presented is comparable to those used in curvature sensors [23,31].

The mathematical computation required to extract the wavefront phase information differs from the variations observed in the state-of-the-art TIE methods [21], as depicted in Bonaque-González et al. [28]. The following equations describe the algorithm behaviour and how to extract the phase of the two images. It should be noted that when we refer to phase, we are indicating the aberrated wavefront, i.e., the points where the electromagnetic waves have the same phase. The algorithm quantifies the differences between a reference wavefront and an aberrated wavefront by measuring the path of light rays at various locations and detecting the optical path difference. SH sensors operate on the same principle, and provide a phase image of the wavefront aberration too.

(4) $$V\left(H_{y,\alpha }\right)\left(x\right)=\overset{x}{\underset{0}{\int }}\;H\left(x,tan\left(\alpha \right)x+y\right)\;for\;\{\begin{array}{c} x\in \left[0,\infty \right)\\ \forall y\in \left[0,\infty \right)\\ \forall \alpha \in \left\{-\pi /2,\pi /2\right\} \end{array}$$

where *H*(*x*,*y*) is a continuous bidimensional function defined for positive values *x, y* and with positive numbers, and *V* the auxiliary transformation of *H*. The character *α* is a variable of *k* discrete angles distributed in the interval [−π/2, π/2], defining a line with y origin and angular separation from the *x* axis equal to *α*.

Another auxiliary function that represents the abscissa axis between two unidimensional continuous functions represented by *f*(*x*) and *g*(*x*) defined for positive values of *x*, represented here as *D,* is described in Equation (5).

(5) $$D\left(f,g\right)\left(x\right)={\mathrm{argmin}}_{x}\left(f\left(x\right)-y\right)-{\mathrm{argmin}}_{x}\left(g\left(x\right)-y\right)\mathrm{for}\;y\in \left[0,\infty \right)$$

The captured images named *I*_1_ and *I*_2_ are different intensity maps, and to recover the phase with our method, Equation (6) is applied.

(6) $${\;\varphi }_{h}\left(x,y\right)=\frac{1}{k}\overset{\alpha =\frac{\pi }{2}}{\underset{\alpha =-\frac{\pi }{2}}{\sum }}cos\left(\alpha \right)D\left(V\left(I_{1\left(y,\alpha \right)}\right),V\left(I_{2\left(y,\alpha \right)}\right)\right)\left(x\right)$$

$${\;\varphi }_{v}\left(x,y\right)=\frac{1}{k}\overset{\alpha =\frac{\pi }{2}}{\underset{\alpha =-\frac{\pi }{2}}{\sum }}sin\left(\alpha \right)D\left(V\left(I_{1\left(y,\alpha \right)}\right),V\left(I_{2\left(y,\alpha \right)}\right)\right)\left(x\right)$$

**Figure 4:**
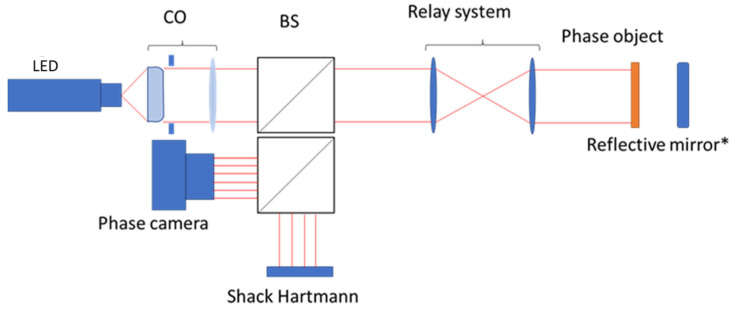
Setup to compare SH sensor with the phase camera. Nomenclature used is described in Figure 1.

It is necessary to apply a factor, represented by Equation (7), to obtain the wavefront phase *φ_o_* in real units (*o* denotes real units). This factor considers the pixel size (s) along with Δz, which represents the propagation distance from the pupil to one of the defocused images. We assume a symmetrical distribution of defocus distances relative to the midpoint (pupil plane) for both defocused images. In our case, the pixel size was chosen in the pupil position, extracted with the calibration target in 11 mm (Figure 4).

(7) $$\varphi_{i}=\varphi_{io}\frac{s^{2}}{2\Delta z}$$

A numerical integration of both gradients is needed to obtain the phase maps [40]. The *k* number of discrete angles to evaluate the images with algorithm is 80, achieving the desired accuracy of our prototype λ/60 without sacrificing the speed of the algorithm; as we checked, more angles do not improve the accuracy when compensated by the execution time, and with more angles it would not be possible to extract the wavefront in real time. In our tests, by adding 120 angles, the accuracy increases to λ/63.

To design our experiment a 4K Sony sensor was chosen, using the half of the height sensor to avoid lens distortions and to limit the pupil of the prototype to 9 mm. It is possible to reach 25 frames per second in the 1200 × 1200 pixels pupils, and 120 with a 650 × 650 pixels pupil. Due to the high amount of data to manage, a Jetson AGX Xavier was chosen to compute the algorithm at a maximum of 25 phase maps per second, allowing real time applications.

The SH sensor was purchased from Thorlabs, model WFS20-14AR, 300 µm pitch, 8–32 taps. The deformable mirror used was DM140A-35-UP01, a 12 × 12 continuous deformable mirror with aluminium coating. The rest of the optical arrangement consists of common lenses and beam splitter 50-50. We chose the ETL, given its speed of movement, a Polight T-Lens Silver [31], and the Sony sensor to obtain a high frame rate. The liquid lens studied with our sensor was an Optotune EL-10-30 [41].

The light source is based in a point-source-type LED (Marktech MTPS9067) emitting at 650 nm, with a window size of 160 um. The beam divergence is adjusted by modifying the distance between the lens and the LED. Note the divergence adjustment is not critical, as any deviation from collimation is seen as a defocus aberration in the wave-front-phase maps that is subtracted from the results. We used achromatic lenses for the entire setup so that the setup would be insensitive to the spectral width of the LED. These achromatic lenses are optimised for the 400–700 nm wavelength range; hence, the same configuration works with the same performance for any LED within the optical range.

## 3. Results and Discussion

### 3.1. Deformable Mirror Study: Shack–Hartman Comparison

This study compares a conventional SH sensor WFS20-14AR with our phase camera. A conventional application of wavefront sensing is adaptive optics, where it is used in a closed loop with a deformable mirror to detect and correct atmospheric turbulences. We have performed a study to evaluate the behaviour of a deformable mirror (DM), characterising the DM errors and the measured error with different actuator distribution. The setup is presented in Figure 4.

**Figure 5:**
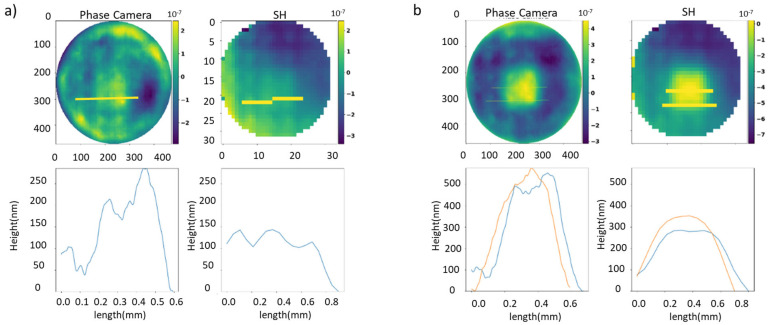
(**a**) 250 nm and (**b**) 500 nm piston of four actuators of the DM for both sensors, together with cross-section profiles indicated by the yellow lines. Different colors in the charts represent a distinct cross-section profile.

We placed an SH sensor in front of an output of the beam splitter, as illustrated in Figure 4. The collimated light pupil was closed to obtain similar diameter in both sensors, lowering the resolution of ours from 1200 × 1200 to 600 × 600. The DM with a 12 × 12 grid actuator matrix provides an actuator pitch of 450 microns, and a maximum height of 3.8 microns. These actuators have a continuous mirror surface, and therefore the shapes have smoothed edges. With the same optical arrangement, multiple shapes were tested. A piston deformation with two heights, 500 nm and 250 nm, is shown in Figure 5. It is possible to distinguish between the different heights of both actuators, and in the 250 nm piston our sensor demonstrates a more dynamic range. The difference between the two adjacent actuators due to the variation in the applied voltage is observed in both pistons. The wavefront figures for both sensors have not been corrected for polynomial tip, slope, or trend errors, causing the graphs to start with non-zero values.

**Figure 6:**
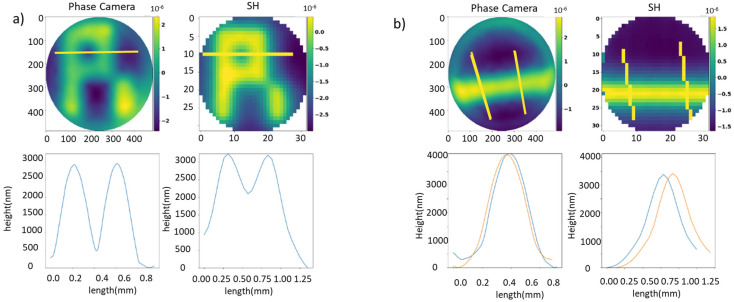
(**a**) R shape depicted with the deformable mirror; phase camera allows for detection of the actuator positioned at 0, opposite to the SH sensor. (**b**) Line in the DM measured at different zones. Different colors in the charts represent a distinct cross-section profile.

In the next example, an R shape was captured using the phase camera and the SH, as shown in Figure 5a, along with the profiles corresponding to the yellow lines indicated on the phase images. From the profiles, it is evident that our camera with higher lateral resolution can accurately register the height step between actuators, unlike the SH sens, or which fails to describe the complete jump and detects an incorrect intermediate height. This example highlights how an increase in lateral resolution not only enables detection of high-frequency details but can also improve the accuracy of phase images along the *Z*-axis, depending on the spatial characteristics of the analyzed sample in Figure 6a.

The measurements obtained by both sensors are highly accurate, as evidenced by the consistency in the height readings obtained from multiple measurements on the same target, as illustrated in Figure 6b. It should be noted that the differences observed are in the same order as the DM internal voltage gradient, as explained later in this section.

**Figure 7:**
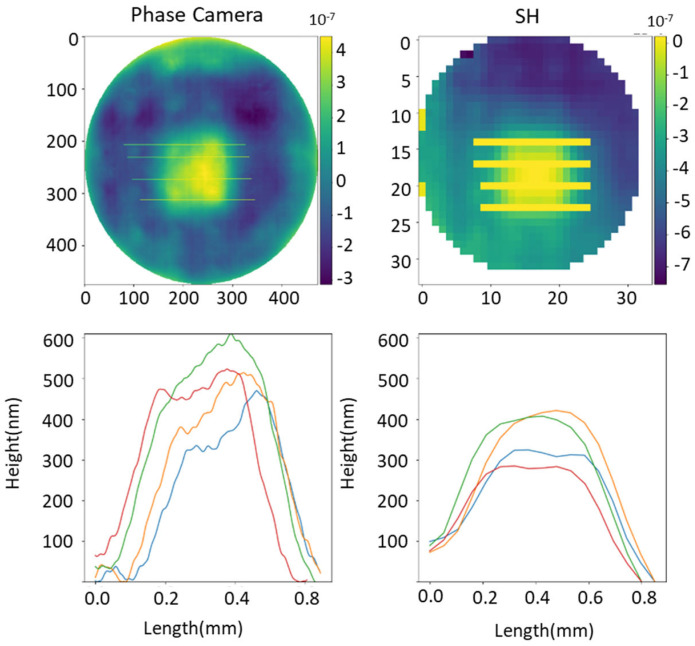
Study of heights at different positions of a piston DM shape. Measures chosen to evaluate the RMS (root mean square) of SH and the presented sensor. Both sensors exhibit distinct responses in the behaviour of the actuators. Different colors in the charts represent a distinct cross-section profile.

We have made a detailed analysis of the DM behaviour. This analysis involves studying the variability between actuators and hence to assess the performance when moving different actuators the same distance apart. Figure 7 reveals that both sensors detect different behaviours between the actuators. The enhanced resolution of our camera allows us to detect a trend, where the actuators on the left, of length 0.2 mm, are positioned below the target point (500 nm), which was consciously achieved in the length 0.5 mm. In contrast, due to the limited resolution of SH, it is difficult to reach the same conclusions in this case.

Table 1 presents the results of an accuracy and repeatability investigation conducted on different shapes on the DM, for both the SH sensor and our phase camera. The table includes various DM shapes for four selected targets: line 4.0 µm, piston 525 nm, R 2.9 µm, and defocus 2.9 µm. The table comprises two evaluation metrics, the root mean square (RMS) and the standard desviation (STD). Equation (9) is employed to assess the RMS variation of the real height of the object at different zones of the targets, with *n* being the value of the multiple lines chosen over the wavefront. The root mean square (RMS) values are obtained from the peak-to-valley (P2V) measurements at multiple locations. The P2V is mathematically represented by Equation 8, wherein L denotes the line of selected points, x1, …, xT represent the points within the lines, and $\varphi_{i}$ corresponds to the wavefront extracted from each DM shape. The STD determines the repeatability between different parts of the target. Therefore, the ideal values for RMS and STD are the actual height value and 0, respectively. To mitigate temporal noise in both sensors, a total of five images are captured and then averaged. Subsequently, distinct zones of the target are selected and compared, using RMS. To measure the accuracy, we assume that the height provided by the DM software is the ground truth, and then the accuracy is the absolute difference between the RMS obtained and the provided software value. This software allows the individual control of actuators by setting voltage values, which are directly convertible into metric units.

(8) $$P2V\left(\varphi_{i}\left(L\right)\right)=\underset{x_{1},\ldots x_{T}}{max}\varphi_{i}-\underset{x_{1},\ldots x_{T}}{min}\varphi_{i}\;$$

(9) $$RMS\left(\varphi_{i}\right)=\sqrt{{\left(\frac{1}{n-1}\overset{n}{\underset{i=1}{\sum }}\left(P2V\left(\varphi_{i}\left(L_{i}\right)\right)-P2V\left(\varphi_{i}(L_{0}\right))\right)\right)}^{2}}\;$$

Table 1 demonstrates that the phase camera presented is more accurate than the SH sensor in the DM shapes. The SH presents more repeatability in the defocused shape case, which is a low-order frequencies wavefront.

**Figure 8:**
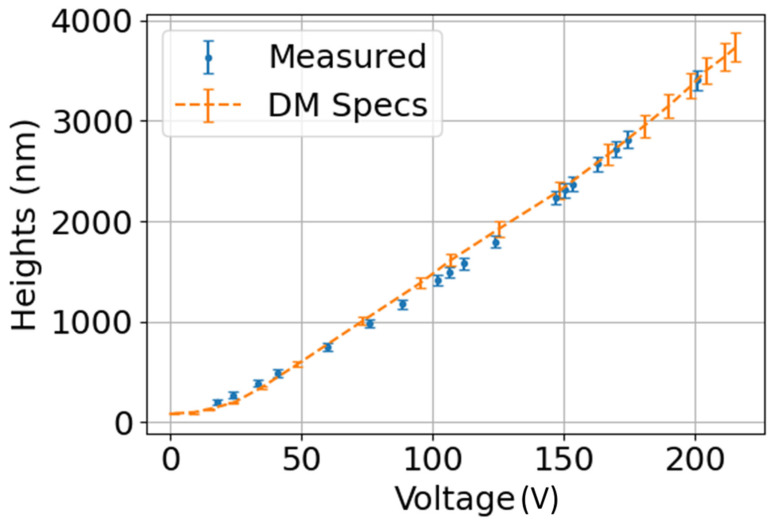
Deformable-mirror study comparison with the measured values of the proposed apparatus. Volt unit in the abscissa; the DM used allows from 0 to 220 volts.

A study was conducted to check the difference between the shapes provided by the deformable mirror and those obtained by our sensor, by measuring different actuator voltages of a piston at each position. We estimated the standard deviation of the heights using the characterisation of the deformable mirror provided by the manufacturer, converting the voltage variations iton nanometers; we compared these with our measurements in Figure 8.

The validity of the ETL optical setup and algorithm was established by verifying our device measurements against the SH, which is considered a standard in the field. The limitations of the SH sensors become apparent in scenarios where high lateral resolution is critical. This is because the minimum change in width or height depends on the number and size of the microlenses. On the other hand, by capturing information at the sensor resolution with the estimated pixel size, our camera can detect higher frequency changes, as demonstrated in Figure 5a. Our design can detect objects with lower phase than SH, as shown in Figure 6. This allows for measurements of up to 250 nm of piston. Comparing deformable-mirror characterisation with the manufacturer’s specifications is critical to determine the precision and repeatability of our system. Figure 8 demonstrates that the measurements acquired by the phase camera are within the standard deviation range supplied by the deformable mirror, indicating that objects with an unknown phase can be measured.

### 3.2. Lens Characterisation

An important application of the wavefront sensor is optics characterisation. Quality lens evaluation is mandatory in the field of optics. A set of multiple lenses was tested to study the different capabilities of the proposed camera. Figure 8 displays fixed-dioptre astigmatism from different angles. It is possible to observe the same dioptre astigmatism from different angles. To determine the angles, we decomposed our phase image into low-order Zernike polynomials and utilised the astigmatism indices, similar to the state-of-the art approach used in [42]. We rounded the obtained angles to the nearest value, to avoid fractional numbers.

For these measurements, and the rest of the examples shown in this article, the SH was removed, and therefore the maximum pupil diameter, 1200 × 1200 pixels, was used.

Taking advantage of the high-speed capture time of our camera, we captured 50 images for each astigmatism. From these images, we obtained the typical deviation over peak-to-valley (P2V) values, which are presented in Table 2 for the case of 1 dioptre of astigmatism with a 6 mm pupil diameter. In this case, the P2V represents the deformation of the lens. To obtain it, Equation (8) was used, but using all the pixels inside the image, obtaining a mean value over the 50 images. The P2V in Table 2 estimates the real accuracy; the theoretical P2V of the lens is 2 × 10^−6^ m. The standard deviation (STD) reflects the variation among the images, serving as a measure of their repeatability.

**Table 2:** P2V and STD in metres, obtained from a batch of 50 images, each of different astigmatism, with angles ranging from 0 to 75, as in Figure 9. The ideal P2V value is 2 µm, and STD must return to 0.

Angle	0	15	30	45	60	75
Target P2V	2 µm	2 µm	2 µm	2 µm	2 µm	2 µm
P2V	2.03 µm	2.12 µm	1.98 µm	2.22 µm	2.07 µm	1.96 µm
STD	0.0182 µm	0.0156 µm	0.0160 µm	0.0131 µm	0.0151 µm	0.0091 µm

This example demonstrates the versatility of our camera for characterising different types of lenses using a compact system, which can be easily adapted to different configurations and experimental conditions. Table 2 shows that the peak-to-valley results are very similar for each angle. The pupil diameter of the images taken was 6 mm and 1 dioptre astigmatism, and the peak-to-valley measurement of the image is 2µm. In this case, a typical laboratory environment was utilised without considering air quality control measures, which could result in a higher standard deviation due to airborne contaminants present in the room. There are several studies of measurement and characterisation of lenses using the wavefront phase, which focuses on obtaining the low-order Zernike polynomials (generally the first 16) [43]. Our characterisation demonstrates the ability to measure various astigmatism angles and multiple dioptres, as well as high frequencies within the lens. This allows us to extract high-order Zernike polynomials or apply high-pass filters to evaluate small or localised deformations.

### 3.3. Tunable Lens in Motion

**Figure 10:**
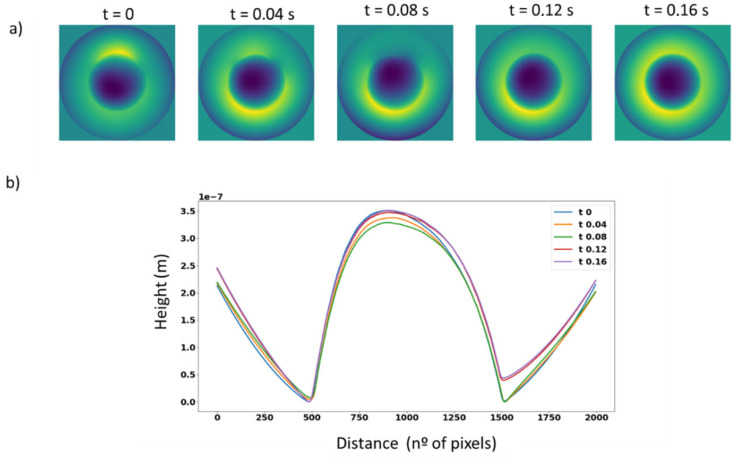
(**a**) 5 consecutives phase maps of a liquid lens in movement. (**b**) Cross-sections of the liquid lens centre.

The behaviour of a liquid lens was characterised demonstrating the real-time capture and processing of our phase camera. Five consecutive frames are shown in Figure 10a, revealing different heights of defocus and the behaviour of the lens movement, which is not a perfect defocus every time, the profile of each defocus is shown in Figure 10b.

The lens characterised is an Optotune EL-10-30 in the visible range (400–700 nm) moving in square form at 25 Hz. It consists of a liquid lens with two liquids, which varies in shape when different currents are applied over time.

The characterisation process of the liquid lens revealed that it requires a certain amount of time to stabilise and attain specific focus points. This information is useful for considering the hysteresis of the lens and to determine its potential applications and drawbacks. The behaviour of the liquid lens can be evaluated by varying the direction of movement and increasing or decreasing dioptres to determine its stability. In this case, it was found that increasing dioptres results in greater focus repeatability, which can be useful in developing algorithms for automatic focus, one of the main applications of ETL [44].

## 4. Conclusions

This work presents the design of a highly versatile phase camera that can be easily adapted for use in various experimental settings without the need for significant modifications to the optical system. The limitations of the ETL have been thoroughly characterised and addressed by software correction, and the optical setup has been optimised to achieve maximum displacement. This adaptable system allows for changes in magnification and other optical adjustments to expand its use in fields such as microscopy, biomedicine, astronomy, and semiconductor metrology. The use of ETLs can be valuable for obtaining the wavefront of an object, solving the limitations outlined in the current literature, such as magnification correction. By integrating this technology into a basic optical setup, several advantages can be gained, including the effortless use of different propagation distances, eliminating the need for a stage to capture multiple images, and avoiding the use of specific prisms, which can reduce sampling resolution. The principal novelty of our camera is the location of the liquid lens, placed before the Fourier plane, in the pupil plane, unlike Zuo et al. [32], who place it in the Fourier plane. The Zuo setup often requires additional control electronics and the physical separation of the lens from the camera, and it involves adapting a camera with a fixed lens, along with a liquid lens specifically designed to capture intensity. This separation delays the synchronisation between the camera and the lens, which is crucial to achieve real-time performance and minimise temporal variability. Therefore, the assembly process becomes more complicated. With this configuration, a pre-assembled liquid-lens capture system can be easily integrated into a phased-imaging system.

We have made a comparison with one of the most widespread phase sensors, and considered a standard in the field, the SH sensor. Our camera has been shown to achieve SH-equivalent results in normal situations, and to improve on SH in situations where lateral resolution is a critical issue. We have seen that better lateral resolution will yield more detail in high frequencies, and better sensibility and accuracy in Z in relatively low frequencies.

Our compact system is suitable for studying and characterising lenses in the visible range of the spectrum, even though, with another design including a different sensor, we could shift to the IR spectral region. Moving forward, metasurfaces are expected to gain prominence, due to their lighter weight and tunability, and their small dimension and complicated spatial design make them ideal candidates for maximum resolution techniques such as the one presented in this article.

We have shown the potential of our camera to work in real-time situations. By providing real-time capture capabilities, this type of camera can be utilised to study a diverse range of samples that exhibit temporal variability in their behaviour. As a result, it is possible to introduce these cameras into fields such as microscopy, where cells undergo structural changes sensitive to phase measurements. Another growing field is real-time laser beam characterisation, where the ability to utilise compact systems that can work in diverse positions and allow for quick interchangeability of resolutions can be an asset.

In summary, our results show that the phase camera developed has a wide range of applications and produces outcomes that are comparable to the SH sensor in low spatial frequencies. By working at the maximum resolution of the sensor with a very small effective pixel size, it becomes possible to achieve the highest level of detail in lateral resolution. This, in turn, enhances the sensitivity to potential high-frequency signals. However, it also increases the Z-sensitivity in intermediate situations and at relatively low frequencies. While such signals can be detected by the SH, their accuracy tends to be lower (Figure 5a).

The future perspectives for phase sensors lie in the development and commercial availability of more instruments that can cover a wider range of fields of application and detect a broader range of spatial frequencies. Such instruments should allow for the simultaneous detection of low- and high-frequency details on the same phase images. In this regard, research studies that combine images captured using techniques like ours, at various propagation distances, represent one of the most important lines of investigation to follow in the coming years.

## Figures and Tables

**Figure 9:**
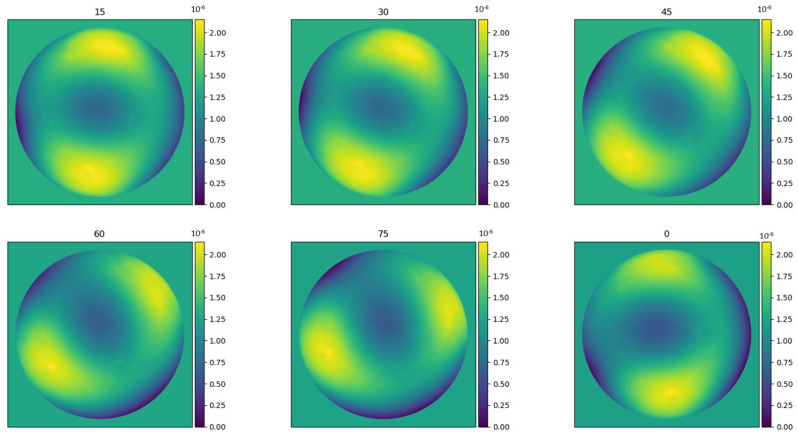
Multiple angles of astigmatism wavefronts, expressed in metres.

**Table 1:** Comparison of the phase camera with the SH sensor on multiple DMs, taking at least 3 measurements in different locations. Root mean square (RMS) and the standard deviation (STD) are presented in meters. The ideal values for RMS and STD are the value of the height and 0, respectively.

Target	R		Line		Piston		Defocus	
DM Value	2.9 µm		4.0 µm		525 nm		2.9 µm	
	RMS	STD	RMS	STD	RMS	STD	RMS	STD
SH	2.47 µm	0.171 µm	3.28 µm	0.0457 µm	364 nm	0.0568 µm	2.42 µm	0.199 µm
Phase Camera	2.79 µm	0.0409 µm	3.97 µm	0.0392 µm	532 nm	0.0508 µm	2.82 µm	0.347 µm

## Data Availability

Data available on request due to restrictions, e.g., privacy or ethical.
